# Ultrafast Self-Assembly of Efficient Blue-Excitable Hybrid Manganese Bromide Microcrystals for Wide-Gamut Display Backlights

**DOI:** 10.3390/molecules31152726

**Published:** 2026-08-06

**Authors:** Huidong Tang, Pengcheng Jiang, Xin Xiong, Xinyi Wen, Jingdan Yan, Simeng Wu, Zhi Wu, Yanqiao Xu, Qing Hu

**Affiliations:** 1 School of Material Science and Engineering, Hunan Institute of Technology, Hengyang 421002, China; hngxy-jiangpc@hnit.edu.cn (P.J.); xwustx@163.com (X.X.); wenxinyi0410@163.com (X.W.); 18699161162@163.com (J.Y.); 18244861189@163.com (S.W.); 2 National Engineering Research Center for Domestic & Building Ceramics, Jingdezhen Ceramic University, Jingdezhen 333001, China; 3 School of Material Science and Engineering, Jingdezhen Ceramic University, Jingdezhen 333001, China; huqing@jci.edu.cn

**Keywords:** hybrid manganese bromide, narrow-band green emission, ultrafast self-assembly, wide gamut displays

## Abstract

Blue-excitable lead-free metal halide microcrystals (MCs) with high efficiency and narrow emission are highly desired for next-generation wide-gamut displays. Herein, an ultrafast self-assembly strategy is developed to prepare hybrid manganese bromide MCs with tunable alkyl-chain lengths. With increasing alkyl-chain length, the emission intensity of the obtained MCs first improves significantly and then deteriorates, accompanied by a gradual narrowing of the full width at half maximum (FWHM) from 46.37 to 41.97 nm. The optimized [(C_2_H_5_)_4_N]_2_MnBr_4_ MCs exhibit strong narrow-band green emission at 518 nm with a FWHM of 43.30 nm and a high photoluminescence quantum yield of 98.70% under 455 nm excitation. This superior emission performance is ascribed to cooperative weak interactions, an appropriate Mn-Mn distance, and highly localized electronic transitions within isolated [MnBr_4_]^2−^ tetrahedra. Moreover, the [(C_2_H_5_)_4_N]_2_MnBr_4_ MCs show moderate thermal stability, retaining 52.03% of their initial emission intensity at 413 K. By integrating [(C_2_H_5_)_4_N]_2_MnBr_4_ with K_2_SiF_6_: Mn^4+^ red phosphors on a blue chip, a white light-emitting diode with a high luminous efficacy of 134.60 lm/W and a wide gamut of 112.6% NTSC is achieved, demonstrating the great promise of ultrafast-assembled hybrid manganese bromides for efficient and wide gamut display backlighting.

## 1. Introduction

Wide-color-gamut displays are attracting increasing attention in high-definition visual technologies, including liquid-crystal displays and mini-LED backlights [1]. In phosphor-converted white light-emitting diodes (WLEDs), the emission bandwidth and emission position of phosphors by blue light excitation directly determine the color purity and achievable color gamut of backlight modules [2]. Narrow-band green phosphors, such as β-SiAlON:Eu^2+^ [3], La_0_._827_Al_11_._9−*x*_Mn*_x_*O_19_._09_ [4], and CsPbBr_3_ [5], together with narrow-band red phosphors, including K_2_SiF_6_: Mn^4+^ [6], K_2_TiF_6_: Mn^4+^ [7] and CsPbI_3_ [8], have been widely explored as color-conversion emitters for wide-gamut display backlights, thereby enabling displays with higher color saturation and improved optical efficiency. However, conventional rare-earth or transition-metal-activated inorganic green phosphors generally require high-temperature solid-state synthesis, which increases energy consumption and limits scalable, low-cost production. In contrast, green-emitting CsPbBr_3_ suffers from the inherent toxicity of Pb and potential instability. Therefore, lead-free green emitters that combine high photoluminescence efficiency, narrow emission, facile synthesis, and good thermal stability are highly desirable for next-generation wide-gamut displays.

Zero-dimensional hybrid Mn^2+^ metal halides, in which isolated [MnX_4_]^2−^ tetrahedra are spatially separated by bulky organic cations, have recently emerged as promising narrow-band green emitters owing to their Mn^2+^ d–d transitions, excitability by blue light, and environmental friendliness [9,10]. For instance, Fan et al. [11] reported (BPTA)_2_MnBr_4_ (BPTA = 3-Bromopropyltrimethylammonium) single crystals (SCs) with a FWHM of 49 nm and a photoluminescence quantum yield (PLQY) of 73.5%, demonstrating that suitable organic-cation selection can regulate the separation and photophysical behavior of the emissive Mn^2+^ centers. Zhang et al. [12] developed C_16_H_38_N_2_MnBr_4_ SCs by structural regulation of the Mn^2+^ coordination environment and Mn···Mn separation, achieving high PLQY of 76% under 460 nm excitation. Duan et al. [13] reported [Et(Ph)_3_P]_2_MnBr_4_ (Et(Ph)_3_P = ethyltriphenylphosphonium) SCs with a 523 nm green emission, a FWHM of 50 nm, and a high PLQY of 94.4% under 450 nm excitation. The corresponding WLED device achieved a luminous efficacy (LE) of 121.3 lm/W and a wide color gamut of 108.7% NTSC. These studies indicate that blue-light excitation can be achieved through diverse organic-cation frameworks, and is modulated by the local Mn^2+^ coordination symmetry, spatial isolation of the inorganic tetrahedra, and nonradiative relaxation processes.

Despite their promising performance as blue-excitable green emitters, hybrid manganese bromides are commonly obtained as single crystals through cooling or antisolvent crystallization [14,15], processes that typically require long growth periods and strict synthetic conditions. To overcome these limitations, mechanochemical method, precipitation method and self-assembly method have been developed for the facile synthesis of hybrid Mn^2+^ metal halide microcrystals (MCs). [16,17,18] For example, Meng et al. [19] synthesized (C_22_H_22_O_2_P)_2_MnBr_4_ MCs by ball-milling, achieving a PLQY of 96.1% under UV light excitation. Zhou et al. [18] reported (C_20_H_20_P)_2_MnBr_4_ MCs prepared via ultrafast self-assembly, which exhibited a PLQY of 93.83% under 450 nm radiation. Nevertheless, the development of highly efficient blue-excitable Mn^2+^ metal halide MCs for high-performance wide-gamut WLEDs remains challenging.

Herein, we report an ultrafast self-assembly strategy for preparing efficient blue-excitable hybrid manganese bromide MCs with different alkyl-chain length, including in [(CH_3_)_4_N]_2_MnBr_4_, [(C_2_H_5_)_4_N]_2_MnBr_4_, and [(C_3_H_7_)_4_N]_2_MnBr_4_. By regulating the alkyl-chain length of the organic cations, the emission behavior and thermal stability of the hybrid manganese bromides are systematically investigated. The optimized [(C_2_H_5_)_4_N]_2_MnBr_4_ MCs exhibit intense narrow-band green emission under blue-light excitation with a near-unity PLQY of 98.70%. The relationships among alkyl-chain length, weak intermolecular interactions, Mn···Mn separation, photoluminescence behavior, and thermal quenching are further discussed. Finally, a WLED device is fabricated by integrating [(C_2_H_5_)_4_N]_2_MnBr_4_ green MCs and commercial K_2_SiF_6_: Mn^4+^ red phosphors with a 450 nm InGaN chip, achieving high LE of 134.60 lm/W and a wide color gamut of 112.6% NTSC. This work provides a simple and efficient strategy for designing lead-free hybrid manganese bromide MCs toward high-efficiency wide-gamut display backlighting.

## 2. Results and Discussion

### 2.1. Synthesis of [(C_2_H_5_)_4_N]_2_MnBr_4_ MCs via Ultrafast Self-Assembly Process

**Figure 1:**
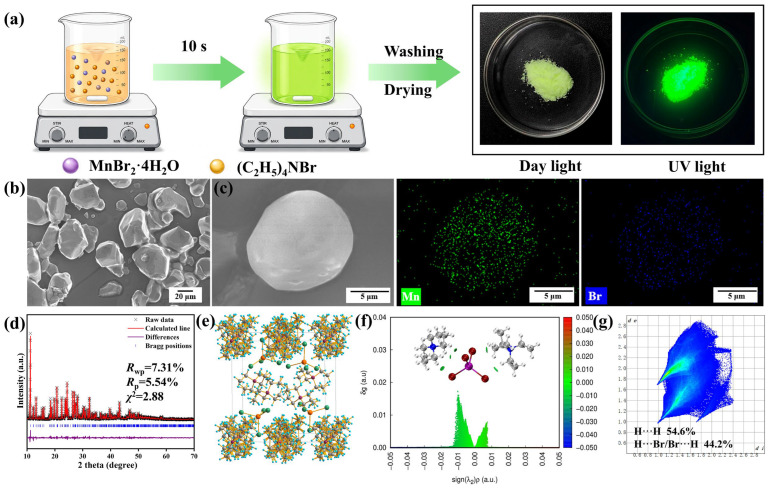
(**a**) Schematic illustration of [(C_2_H_5_)_4_N]_2_MnBr_4_ MCs prepared via an ultrafast self-assembly process, (**b**) SEM image and (**c**) EDS mapping, (**d**) Rietveld patterns, (**e**) crystal structure (Grey spheres (C), Orange spheres (Mn), Green spheres (Br), Magenta spheres (N), and Blue sphere (H); Solid lines indicate chemical bonds), (**f**) scatter diagram of the δ_g_ isosurface vs. sign(λ_2_)ρ, and (**g**) fingerprint pattern of [(C_2_H_5_)_4_N]_2_MnBr_4_ MCs.

[(C_2_H_5_)_4_N]_2_MnBr_4_ MCs were synthesized through an ultrafast self-assembly process, as illustrated in Figure 1a. Briefly, (C_2_H_5_)_4_NBr and MnBr_2_·4H_2_O with a molar ratio of 2.25 were dissolved in 10 mL of ethanol under stirring. A green emissive suspension was immediately observed under UV-light irradiation, indicating the rapid formation of [(C_2_H_5_)_4_N]_2_MnBr_4_ MCs. The as-prepared [(C_2_H_5_)_4_N]_2_MnBr_4_ exhibits an irregular morphology (Figure 1b). The Mn and Br elements with the molar ratio of 1:4.16 are uniformly distributed within the particles by energy-dispersive X-ray spectroscopy (EDS) mapping (Figure 1c), suggesting a slightly Br-rich chemical environment in the obtained MCs.

The diffraction peaks of the MCs prepared by the ultrafast self-assembly process are in good agreement with the simulated pattern of [(C_2_H_5_)_4_N]_2_MnBr_4_ (CCDC 2042083). The Rietveld refinement gives *R*_wp_, *R*_p_, and χ^2^ values of 7.31%, 5.54%, and 2.88, respectively, confirming the reliability of the refined structure (Figure 1d). [(C_2_H_5_)_4_N]_2_MnBr_4_ crystallizes in the tetragonal *P-4(_)2_1_m* space group (No. 113). The refined lattice parameters are a = 13.3703 Å, b = 13.3703 Å, c = 14.4230 Å, V = 2578.313 Å^3^ and Z = 2, as summarized in Table S1. In this structure, the bulky (C_2_H_5_)_4_N^+^ cations separate the [MnBr_4_]^2−^ tetrahedra, giving rise to a large Mn···Mn distance of 9.454 Å.

To understand the ultrafast self-assembly behavior of [(C_2_H_5_)_4_N]_2_MnBr_4_ MCs in ethanol, the interactions between (C_2_H_5_)_4_N^+^ and [MnBr_4_]^2−^ were analyzed using Independent Gradient Model based on Hirshfeld partitioning (IGMH). Figure 1f presents the δ_g_ versus sign(λ_2_)ρ scatter plot and the corresponding isosurfaces of [(C_2_H_5_)_4_N]_2_MnBr_4_. The green regions with sign(λ_2_)ρ values close to zero indicate weak van der Waals interactions. The IGMH results suggest that the interactions between (C_2_H_5_)_4_N^+^ and [MnBr_4_]^2−^ tetrahedra are mainly associated with van der Waals interactions and non-classical C–H···Br hydrogen bonds [20].

Furthermore, Hirshfeld surface analysis and two-dimensional fingerprint plots were used to quantitatively evaluate the intermolecular contacts, as shown in Figure S1 and Figure 1g. Here, *d*_i_ represents the distance from the Hirshfeld surface to the nearest atom inside the surface, whereas *d*_e_ denotes the distance to the nearest atom outside the surface [21]. The fingerprint plots show that H···H contacts account for 54.6% of the total Hirshfeld surface, while H···Br/Br···H contacts contribute 44.2%. These results confirm the presence of abundant H···H and H···Br/Br···H interactions in [(C_2_H_5_)_4_N]_2_MnBr_4_. The weak interactions, particularly the non-classical C–H···Br hydrogen bonds, may play an important role in facilitating the rapid assembly of [(C_2_H_5_)_4_N]_2_MnBr_4_ MCs.

In addition, the Gibbs free energy change (ΔG) for the reaction in ethanol was calculated according to the following equation:

ΔG = ∑ G_product_ − ∑ G_reactant_ (1)

The calculated ΔG is −42.49 kcal/mol (Table S2), which is lower than that reported for the synthesis of (C_20_H_20_P)_2_MnBr_4_ under a similar reaction system [18]. The negative ΔG value indicates that the formation of [(C_2_H_5_)_4_N]_2_MnBr_4_ can proceed spontaneously in ethanol.

### 2.2. PL Properties of Hybrid Manganese Bromide with Different Alkyl-Chain Length

**Figure 2:**
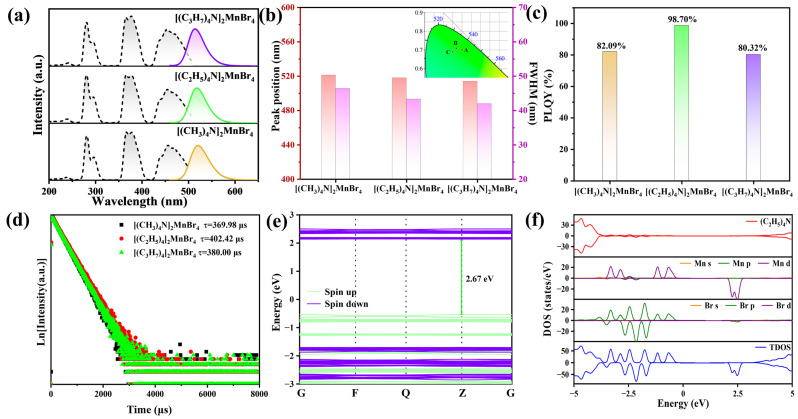
(**a**) PLE and PL spectra, (**b**) emission peak position and FWHM (Inserting the CIE 1931 chromaticity diagram), (**c**) PLQY, (**d**) PL decays of [(CH_3_)_4_N]_2_MnBr_4_, [(C_2_H_5_)_4_N]_2_MnBr_4_, and [(C_3_H_7_)_4_N]_2_MnBr_4_ MCs, (**e**) energy band structure and (**f**) density of states of [(C_2_H_5_)_4_N]_2_MnBr_4_.

The PL excitation (PLE) and PL spectra of [(CH_3_)_4_N]_2_MnBr_4_, [(C_2_H_5_)_4_N]_2_MnBr_4_, and [(C_3_H_7_)_4_N]_2_MnBr_4_ MCs synthesized through an ultrafast self-assembly process are shown in Figure 2a. The excitation spectra of MCs with different alkyl-chain length show similar features, with four distinct excitation bands at 200–250 nm, 250–330 nm, 330–415 nm and 415–510 nm. Among them, the excitation bands in 415–510 nm are relatively intense, indicating effective excitation under blue-light irradiation. Specifically, the excitation peaks at 472, 455, 437, 391, 378 and 365 nm are attributed to the characteristic spin-forbidden d–d transitions of Mn^2+^, corresponding to ^6^A_1_(^6^S) → ^4^T_1_(^4^G), ^6^A_1_(^6^S) → ^4^T_2_(^4^G), ^6^A_1_(^6^S) → [^4^A_1_(^4^G), ^4^E(^4^G)], ^6^A_1_(^6^S) → ^4^T_2_(^4^D), ^6^A_1_(^6^S) → ^4^E(^4^D) and ^6^A_1_(^6^S) → ^4^T_1_(^4^P), respectively. Under 455 nm irradiation, the MCs exhibit strong narrow-band green emission. As the alkyl-chain length increases, the emission maximum of the MCs exhibits a gradual blue shift from 521 nm to 514 nm, accompanied by a narrowing of the FWHM from 46.37 nm of [(CH_3_)_4_N]_2_MnBr_4_ MCs to 41.97 nm of [(C_3_H_7_)_4_N]_2_MnBr_4_ MCs (Figure 2b). Remarkably, [(C_2_H_5_)_4_N]_2_MnBr_4_ MCs show the strongest emission among the prepared MCs, with a PLQY of 98.70% (Figure 2c and Figure S2). The Commission Internationale de l’Éclairage (CIE) chromaticity coordinates of MCs with different alkyl-chain lengths are A (0.20, 0.70) of [(CH_3_)_4_N]_2_MnBr_4_, B (0.17, 0.71) of [(C_2_H_5_)_4_N]_2_MnBr_4_ and C (0.15, 0.69) of [(C_3_H_7_)_4_N]_2_MnBr_4_, respectively, which are close to the NTSC standard green coordinate of (0.21, 0.71), highlighting the potential of these hybrid manganese bromide green emitters for display applications.

To understand the emission mechanism, the adjacent Mn-Mn distance is studied. The Rietveld refinement results of [(C_3_H_7_)_4_N]_2_MnBr_4_ MCs (Figure S3 and Table S3) show the shortest Mn-Mn distance of 9.655 Å. Combing with the previously reported [22,23], the shortest Mn-Mn distances in the green MCs increase in the order of [(CH_3_)_4_N]_2_MnBr_4_ < [(C_2_H_5_)_4_N]_2_MnBr_4_ < [(C_3_H_7_)_4_N]_2_MnBr_4_, indicating that elongation of the alkyl chain leads to greater spatial separation of the isolated [MnBr_4_]^2−^ tetrahedra. As the alkyl-chain length of the organic precursor increases, the enlarged Mn-Mn distance weakens the interactions and excitation-energy migration among adjacent [MnBr_4_]^2−^ tetrahedra, thereby suppressing concentration quenching and contributing to the efficient green emission [24,25,26]. Nevertheless, an overly large Mn···Mn separation may introduce nonradiative channels that consequently decrease the emission intensity [27].

The photoluminescence emission intensity is not only related to the Mn-Mn distance but is also strongly affected by defect states. Figure 2d shows the PL decay curves of MCs with different alkyl-chain lengths. These decay curves can be fitted by a single-exponential function, as follows: [22]

*I*(*t*) = *I*_0_ + A exp(−*t*/*τ*) (2)

where the *I*(t) and *I*_0_ are the fluorescence intensity at time *t* and 0, respectively. The PL decay curves of MCs can be well fitted by a single-exponential function, indicating that the emission predominantly originates from a single type of emissive center, which is consistent with Mn^2+^-centered d–d transitions. The calculated lifetimes of the [(CH_3_)_4_N]_2_MnBr_4_, [(C_2_H_5_)_4_N]_2_MnBr_4_, and [(C_3_H_7_)_4_N]_2_MnBr_4_ MCs are 369.95, 402.42 and 380.00 μs, respectively, confirming that the emission center arises from the d–d transitions of Mn^2+^.

The radiative and nonradiative decay rates from the PLQYs and PL lifetimes were further calculated using *k*_r_ = Φ/τ and *k*_nr_ = (1 − Φ)/τ. The calculated *k*_nr_ values are approximately 4.84 × 10^2^, 3.23 × 10^1^, and 5.18 × 10^2^ s^−1^ for the [(CH_3_)_4_N]_2_MnBr_4_, [(C_2_H_5_)_4_N]_2_MnBr_4_, and [(C_3_H_7_)_4_N]_2_MnBr_4_, respectively. The corresponding radiative rates are approximately 2.22 × 10^3^, 2.45 × 10^3^, and 2.11 × 10^3^ s^−1^. Therefore, the [(C_2_H_5_)_4_N]_2_MnBr_4_ exhibits not only the highest radiative rate but, more importantly, a nonradiative decay rate that is more than one order of magnitude lower than those of the [(CH_3_)_4_N]_2_MnBr_4_ and [(C_3_H_7_)_4_N]_2_MnBr_4_. These results demonstrate that near-unity PLQY of [(C_2_H_5_)_4_N]_2_MnBr_4_ arises from effective suppression of nonradiative deactivation.

Figure S4a,b presents the PLE contour maps and selected PLE spectra of [(C_2_H_5_)_4_N]_2_MnBr_4_ MCs, monitored at different emission wavelengths. The PLE spectra of MCs show similar profiles at different emission wavelengths, and no obvious shift in the excitation peaks is observed. In addition, the PL contour maps and selected PL spectra of [(C_2_H_5_)_4_N]_2_MnBr_4_ MCs under different excitation wavelengths are shown in Figure S4c,d. The PL spectra of phosphors retain similar spectral shapes under different excitation wavelengths, and their emission peaks exhibit no noticeable shift. Temperature-dependent PL decay measurements further were studied, as shown in Figure S5. The PL decay curves can be approximately fitted using a single-exponential function with fitted lifetimes of 391.03, 393.00, 402.42, and 412.09 μs at 103, 203, 303, and 403 K, respectively. These results demonstrate that the emission of [(C_2_H_5_)_4_N]_2_MnBr_4_ MCs originates from the radiative d–d transitions of Mn^2+^ rather than defect-related emission.

The effect of the alkyl-chain length on the photoluminescence properties of the green MCs was further discussed based on crystal-field theory for the 3d^5^ electronic configuration of Mn^2+^, as shown in Figure S6. These results show that the crystal-field strength follows the order of [(CH_3_)_4_N]_2_MnBr_4_ > [(C_2_H_5_)_4_N]_2_MnBr_4_ > [(C_3_H_7_)_4_N]_2_MnBr_4_, which indicates that the crystal-field strength gradually decreases with increasing alkyl-chain length. The decrease in crystal-field strength with increasing alkyl-chain length is consistent with the observed blue shift in Mn^2+^ emission. Meanwhile, the reduced Racah parameter B suggests enhanced Mn–Br covalency and a stronger nephelauxetic effect, which may contribute to the narrowing of the emission bandwidth.

To gain insight into the photoluminescence mechanism of [(C_2_H_5_)_4_N]_2_MnBr_4_, Density functional theory (DFT) calculations were performed to investigate its band structure and electronic structure. Figure 2e,f show the calculated band structure and density of states of [(C_2_H_5_)_4_N]_2_MnBr_4_ respectively. [(C_2_H_5_)_4_N]_2_MnBr_4_ exhibits a direct bandgap of 2.67 eV which is close to the optical bandgap of 2.37 eV estimated from the absorption spectrum (Figure S7). Compared with [(CH_3_)_4_N]_2_MnBr_4_, [(C_2_H_5_)_4_N]_2_MnBr_4_ possesses a smaller bandgap, which is beneficial for facilitating radiative recombination. In addition, the energy bands near the valence-band maximum (VBM) and conduction-band minimum (CBM) in the Brillouin zone are relatively flat, which can be attributed to the structural isolation of the [MnBr_4_]^2−^ tetrahedra by bulky organic cations. the VBM of [(C_2_H_5_)_4_N]_2_MnBr_4_ is mainly composed of hybridized Mn 3d and Br 4p orbitals, whereas the CBM is dominated by Mn 3d orbitals. The orbital hybridization between Br 4p and Mn 3d through Mn–Br coordination helps relax the spin-forbidden selection rule of the Mn^2+^ d–d transitions [12]. Moreover, the (C_2_H_5_)_4_N^+^ cationic orbitals exhibit a large bandgap and show negligible coupling with Mn and Br orbitals, indicating that the emission of [(C_2_H_5_)_4_N]_2_MnBr_4_ mainly originates from the d–d transitions within the [MnBr_4_]^2−^ tetrahedra. The electronic isolation of the [MnBr_4_]^2−^ tetrahedra further promotes the highly localized nature of these d–d transitions.

### 2.3. Stability of Hybrid Manganese Bromide MCs

**Figure 3:**
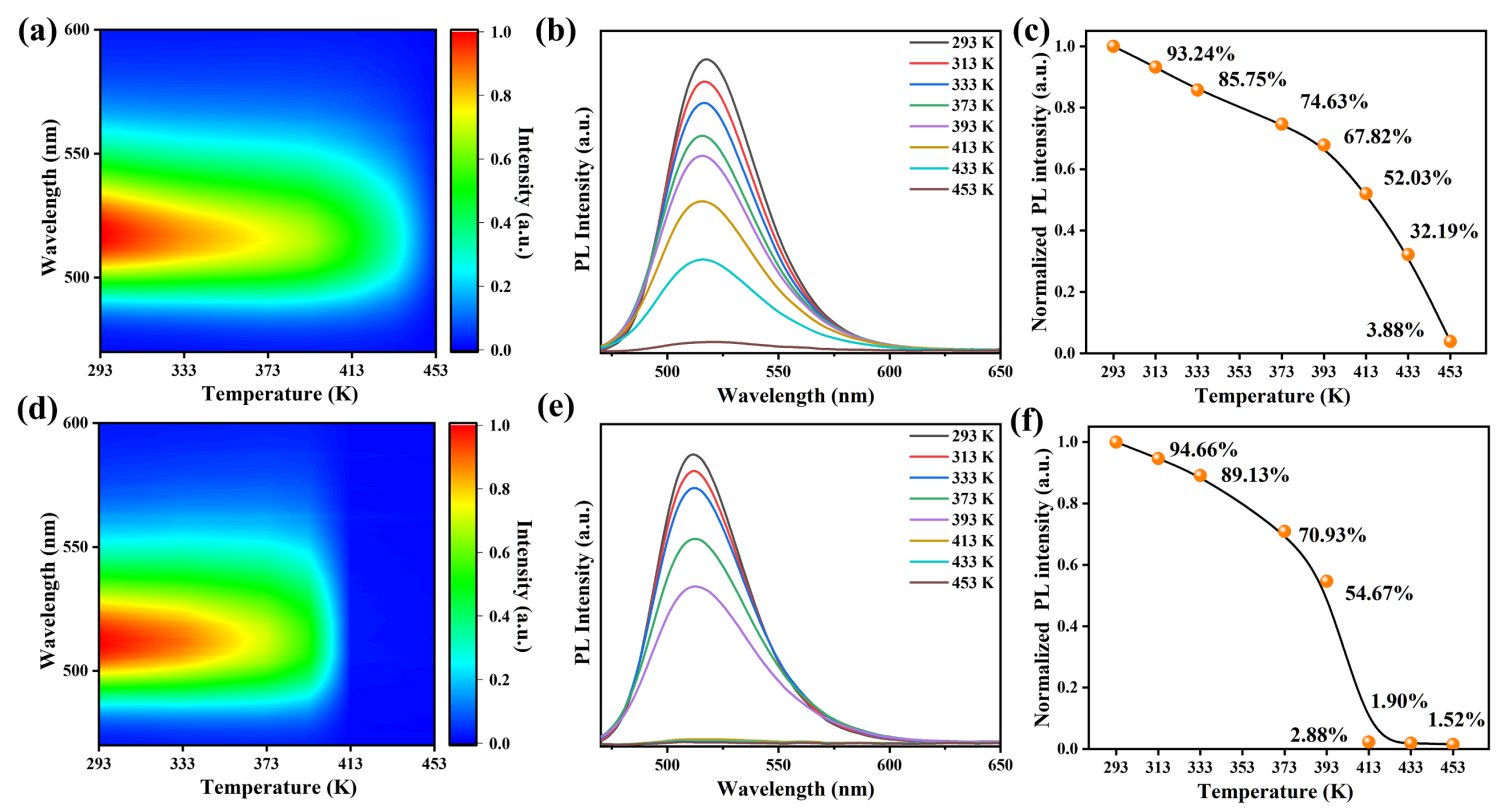
Pseudocolor PL contour maps of (**a**) [(C_2_H_5_)_4_N]_2_MnBr_4_ and (**d**) [(C_3_H_7_)_4_N]_2_MnBr_4_ MCs measured at different temperatures, temperature-dependent PL spectra of (**b**) [(C_2_H_5_)_4_N]_2_MnBr_4_ and (**e**) [(C_3_H_7_)_4_N]_2_MnBr_4_ MCs, evolution of emission intensity of (**c**) [(C_2_H_5_)_4_N]_2_MnBr_4_ and (**f**) [(C_3_H_7_)_4_N]_2_MnBr_4_ with different temperature.

Thermal stability of [(C_2_H_5_)_4_N]_2_MnBr_4_ and [(C_3_H_7_)_4_N]_2_MnBr_4_ MCs were studied by temperature-dependent PL measurements, as shown in Figure 3. PL maps and spectra of [(C_2_H_5_)_4_N]_2_MnBr_4_ and [(C_3_H_7_)_4_N]_2_MnBr_4_ MCs reveal a progressive decrease in emission intensity with increasing temperature, owing to enhanced thermally activated nonradiative recombination. Meanwhile, both MCs show a gradual blue shift in the emission peak upon heating, which is associated with thermally induced lattice expansion. Notably, [(C_2_H_5_)_4_N]_2_MnBr_4_ MCs can retain 52.03% of its initial PL intensity at 413 K relative to that at 293 K (Figure 3c), which is inferior to commercial β-SiAlON:Eu^2+^ and NaK_2_Li[Li_3_SiO_4_]_4_:Eu [28,29]. However, [(C_3_H_7_)_4_N]_2_MnBr_4_ MCs retain only 2.88% (Figure 3f), indicating pronounced thermal quenching. In comparison with [(CH_3_)_4_N]_2_MnBr_4_ [22], these results demonstrate that elongation of the organic alkyl chain leads to a gradual deterioration in the thermal stability of hybrid Mn^2+^ bromide MCs.

To clarify the correlation between organic alkyl-chain length and thermal stability, Hirshfeld surface analysis of [(CH_3_)_4_N]_2_MnBr_4_ and [(C_3_H_7_)_4_N]_2_MnBr_4_ was carried out, as shown Figure S8 and Table S4. The main intermolecular contacts in [(CH_3_)_4_N]_2_MnBr_4_ are H···H and H···Br/Br···H interactions, accounting for 43.4% and 56.1%, respectively. For [(C_3_H_7_)_4_N]_2_MnBr_4_, the corresponding contributions are 64.1% and 33.7%, respectively. These results indicate that elongation of the organic alkyl chain substantially reduces the proportion of H···Br/Br···H contacts from 56.1% to 33.7%, weakening the hydrogen-bonding interactions in the crystal lattice and consequently deteriorating the thermal stability.

The influence of increasing alkyl-chain length on the spatial arrangement of the isolated [MnBr_4_]^2−^ tetrahedra, the organic–inorganic intermolecular interactions, and the resulting photoluminescence properties are summarized in Figure S9. With increasing alkyl-chain length, the spatial separation between neighboring tetrahedra increases, whereas the contribution of H···Br/Br···H contacts decreases from 56.1% to 33.7% The enlarged Mn···Mn separation weakens Mn^2+^–Mn^2+^ coupling and suppresses excitation-energy migration. In parallel, the gradual decrease in crystal-field strength accounts for the observed blue shift and narrowing of the Mn^2+^ emission band. The PLQY exhibits a non-monotonic dependence on alkyl-chain length, increasing from 82.09% to 98.70% and then decreasing to 80.32%. The near-unity PLQY of [(C_2_H_5_)_4_N]_2_MnBr_4_ MCs is attributed to an appropriate Mn···Mn separation, organic–inorganic interactions and local crystal-field strength, as well as fewer nonradiative relaxation.

### 2.4. The Application of WLED Based on [(C_2_H_5_)_4_N]_2_MnBr_4_ MCs

**Figure 4:**
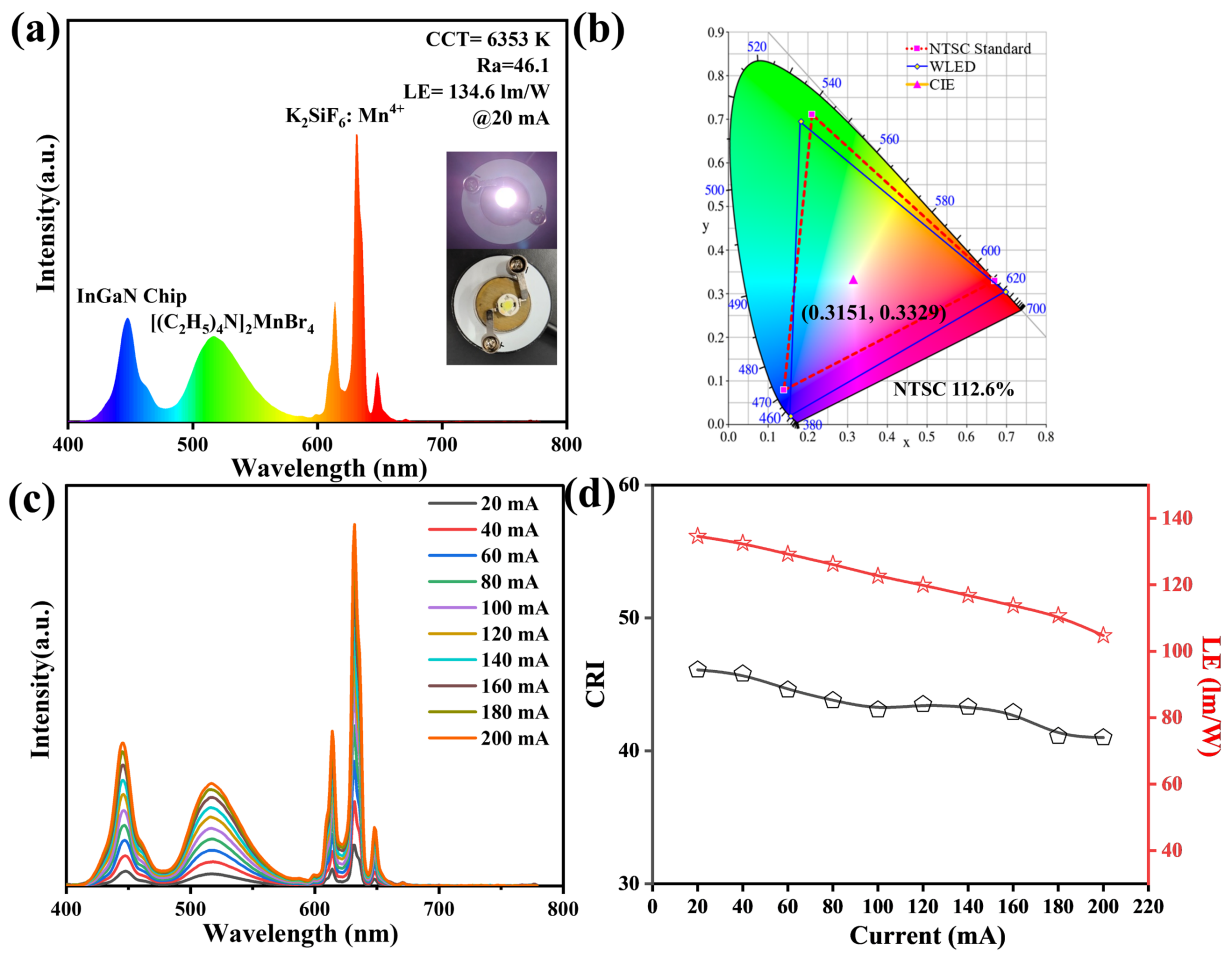
(**a**) EL spectrum of the WLED fabricated using [(C_2_H_5_)_4_N]_2_MnBr_4_ green MCs and K_2_SiF_6_: Mn^4+^ red phosphors (Inserting the photographs of the WLED devices operated at 0 mA and 20 mA), (**b**) CIE 1931 chromaticity diagram, (**c**) EL spectra of the WLED under different driving currents, (**d**) CRI and luminous efficacy as functions of driving current.

The as-prepared [(C_2_H_5_)_4_N]_2_MnBr_4_ MCs, serving as the green emitter, were combined with commercial K_2_SiF_6_: Mn^4+^ red phosphors and integrated with a 450 nm InGaN LED chip to fabricate a WLED device. Figure 4a shows the EL spectrum of the WLED recorded at a driving current of 20 mA. The device exhibits bright white emission with CIE chromaticity coordinates of (0.3151, 0.3329), which are close to the standard white-light coordinates of (0.33, 0.33). The WLED also shows a color rendering index (*R*_a_) of 46.1 and a correlated color temperature (CCT) of 6353 K. Benefiting from the efficient green emission of [(C_2_H_5_)_4_N]_2_MnBr_4_ MCs under blue-light excitation, the fabricated WLED achieves a high LE of 134.60 lm/W, outperforming many reported WLEDs based on hybrid manganese halides (Table S5), such as 117.17 lm/W of (C_20_H_20_P)_2_MnBr_4_ based on WLED [18], 121.3 lm/W of [Et(Ph)_3_P]_2_MnBr_4_ based on WLED [13], and C_16_H_38_N_2_MnBr_4_ based on 133 lm/W of WLED [12]. As shown in Figure 4b, the gamut of the [(C_2_H_5_)_4_N]_2_MnBr_4_-based WLED is calculated to be 112.6% of the NTSC standard, confirming its wide-gamut emission characteristics and promising potential for LCD backlight applications. Furthermore, the current-dependent EL performance of the [(C_2_H_5_)_4_N]_2_MnBr_4_-based WLED was investigated, as shown in Figure 4c. The EL spectral profile remains nearly unchanged as the driving current increases, while the emission intensity gradually increases. When the current is increased to 200 mA, the LE remains as high as 104.70 lm/W (Figure 4d), indicating the favorable current tolerance of the [(C_2_H_5_)_4_N]_2_MnBr_4_-based WLED device.

## 3. Experiment and Characterization

### 3.1. Materials

Manganese (II) bromide tetrahydrate (MnBr_2_·4H_2_O, 99%, Aladdin, Shanghai, China), tetramethylammonium bromide ((CH_3_)_4_NBr, 98%, Aladdin, Shanghai, China), tetraethylammonium bromide ((C_2_H_5_)_4_NBr, 98%, Aladdin, Shanghai, China), tetrapropylammonium bromide ((C_3_H_7_)_4_NBr, 98%, Aladdin, Shanghai, China), and ethanol (C_2_H_5_OH, ≥99.7%, Sinopharm, Beijing, China) were used without further purification.

### 3.2. Synthesis of Hybrid Manganese Bromide MCs via Ultrafast Self-Assembly Method

For the synthesis of [(C_2_H_5_)_4_N]_2_MnBr_4_ MCs, 2.25 mmol of (C_2_H_5_)_4_NBr and 1.00 mmol of MnBr_2_·4H_2_O were dissolved in 10 mL of ethanol under stirring for 500 rpm. A green suspension formed rapidly within 10 s at room temperature. The suspension was collected, washed thoroughly with ethanol, and dried at 60 °C for 6 h to obtain [(C_2_H_5_)_4_N]_2_MnBr_4_ MCs. [(CH_3_)_4_N]_2_MnBr_4_ and [(C_3_H_7_)_4_N]_2_MnBr_4_ MCs were synthesized using the same procedure, except that (C_2_H_5_)_4_NBr was replaced with (CH_3_)_4_NBr or (C_3_H_7_)_4_NBr, respectively.

### 3.3. Preparation of WLED Device

To fabricate the WLED device, [(C_2_H_5_)_4_N]_2_MnBr_4_ MCs, K_2_SiF_6_: Mn^4+^ phosphors, and UV-curable silicone were thoroughly mixed at a mass ratio of 1:0.8:2. The obtained phosphor–silicone composite was subsequently coated onto a 450 nm blue InGaN chip and cured under UV irradiation for 15 min.

### 3.4. Characterization

The morphology and elemental distributions of MCs were characterized by scanning electron microscopy (SEM, ZEISS Gemini 300, Oberkochen, Germany) equipped with EDS. The structure data of hybrid manganese bromide MCs were recorded by powder X-ray diffraction (XRD, Bruker D8 Advance, Billerica, MA, USA) and Rietveld refinement by GSAS 3.4.5 package with the EXPGUI interface [30]. PL properties of MCs were measured using a fluorescence spectrometer (Edinburgh FS5 v2, Scotland, UK). Absorption spectrum of [(C_2_H_5_)_4_N]_2_MnBr_4_ MCs were recorded on a UV–vis spectrophotometer (PerkinElmer Lambda 750, Waltham, MA, USA). The electroluminescence (EL) characteristics of the LED devices were evaluated using a spectroradiometer (Hopocolor HP8000, Hangzhou, China).

### 3.5. Absolute PLQY Measurements

The absolute PLQY of the hybrid manganese bromide MCs were measured using an Edinburgh Instruments FS5 v2 spectrofluorometer equipped with an SC-30 integrating-sphere module. The PLQY was calculated by

(3) $$PLQY=\frac{E_{s}-E_{r,\mathrm{scaled}}}{S_{r}-S_{s}}$$

where *E*_s_ is the integrated emission intensity of the sample, *E*_r,scaled_ is the scaled reference background within the emission region, and Sr and Ss are the integrated excitation-scattering intensities of the reference and sample, respectively.

### 3.6. Theoretical Calculations

Noncovalent interactions in [(C_2_H_5_)_4_N]_2_MnBr_4_ were analyzed using the IGMH implemented in Multiwfn 3.8 [31] and the generated isosurfaces were rendered with VMD 1.9.3 [32]. Hirshfeld surface analysis of the hybrid manganese bromide MCs was performed using CrystalExplorer21 [33]. DFT calculations were further carried out with CASTEP 6.0 to obtain the electronic band structure and bandgap of [(C_2_H_5_)_4_N]_2_MnBr_4_. A periodic structural model with the composition C_64_H_160_N_8_Mn_4_Br_16_, comprising a total of 252 atoms, was constructed for the calculations. The Brillouin zone was sampled using a 2 × 2 × 2 Monkhorst–Pack *k*-point mesh. The exchange–correlation interactions were described within the generalized gradient approximation (GGA) using the Perdew–Burke–Ernzerhof (PBE) functional [34,35]. Spin polarization was included to account for the magnetic characteristics of Mn^2+^ ions. The plane-wave cutoff energy, self-consistent-field convergence criterion and maximum residual force were set to 500 eV, 1.0 × 10^−5^ eV and 0.02 eV Å^−1^, respectively.

## 4. Conclusions

In summary, blue-light-excitable hybrid manganese bromide MCs were developed through an ultrafast self-assembly strategy. The alkyl-chain length of the organic cation plays an important role in regulating the optical properties and thermal stability of the hybrid manganese bromides. With increasing alkyl-chain length, the emission peak gradually blue-shifts from 521 nm to 514 nm, while the FWHM narrows from 46.37 to 41.97 nm. Among the synthesized samples, [(C_2_H_5_)_4_N]_2_MnBr_4_ MCs have the optimal performance, with a near-unity PLQY of 98.70%, which can be attributed to appropriate Mn···Mn separation, organic–inorganic interactions, and suppression of nonradiative relaxation. The optimized MCs retain 52.03% of their initial PL intensity at 413 K. A WLED fabricated by combining [(C_2_H_5_)_4_N]_2_MnBr_4_ green MCs with K_2_SiF_6_: Mn^4+^ red phosphors on a 450 nm InGaN chip achieves an LE of 134.60 lm/W and a wide color gamut of 112.6% NTSC. The as-prepared WLED has excellent current tolerance, with its electroluminescence intensity increasing monotonically as the driving current rises from 20 mA to 200 mA, while retaining a high LE of 104.70 lm/W at 200 mA. This work establishes clear relationships among alkyl-chain length, Mn···Mn separation, intermolecular interactions, and luminescence performance, providing valuable design principles and a scalable ultrafast self-assembly strategy for developing efficient, thermally stable, lead-free hybrid manganese halide emitters for wide-gamut displays.

## Data Availability

The data presented in this study are available on request from the corresponding authors.

## Supplementary Materials

The following supporting information can be downloaded at: https://www.mdpi.com/article/10.3390/molecules31152726/s1. Refs. [36,37,38] are cited in Supplementary Materials.
