# Supplementary material for: Ultrafast Self-Assembly of Efficient Blue-Excitable Hybrid Manganese Bromide Microcrystals for Wide-Gamut Display Backlights
Source: Molecules. 2026 Aug 6;31(15):2726. doi: 10.3390/molecules31152726 (PMC13468644; doi:10.3390/molecules31152726)
Supplement: Supplementary file 1 [file molecules-31-02726-s001.zip › molecules-4431682-supplementary.pdf]

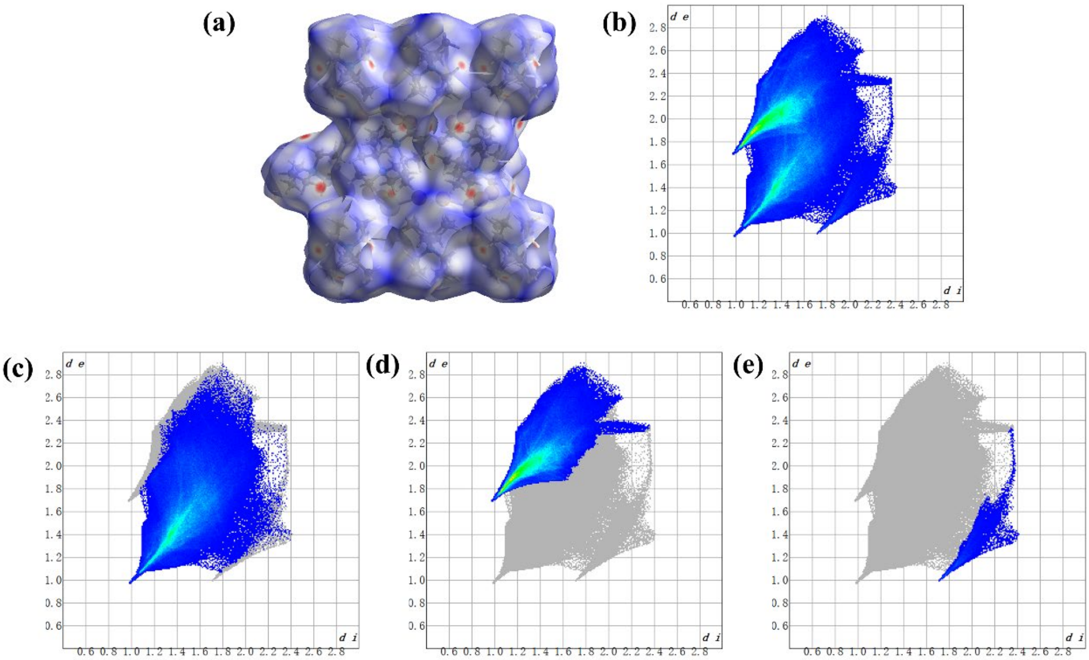

Figure S1. Hirshfeld surface (a) and fingerprint plots (b) of $[(C_2H_5)_4N]_2MnBr_4$ MCs: H···H (c), H···Br (d) and Br···H (e) interactions.

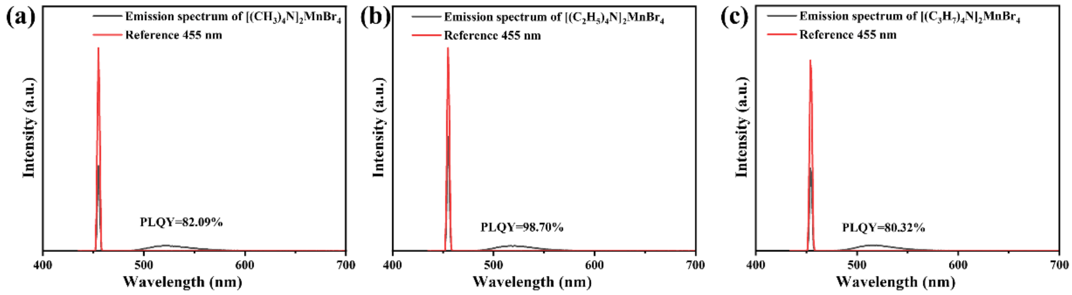

Figure S2. PLQY of (a) $[(CH_3)_4N]_2MnBr_4$, (b) $[(C_2H_5)_4N]_2MnBr_4$, and (c) $[(C_3H_7)_4N]_2MnBr_4$ MCs.

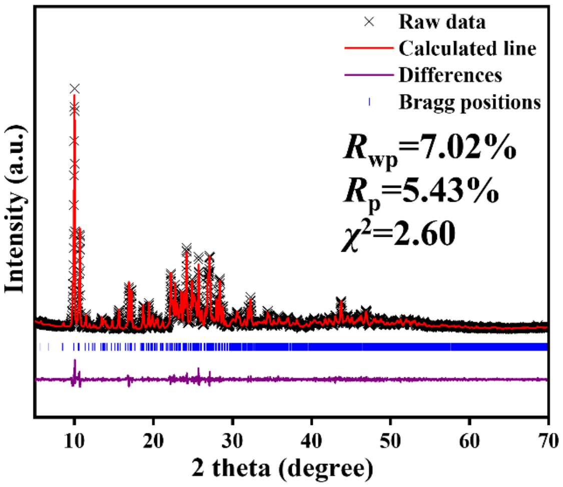

Figure S3. Rietveld patterns of $[(C_3H_7)_4N]_2MnBr_4$ MCs.

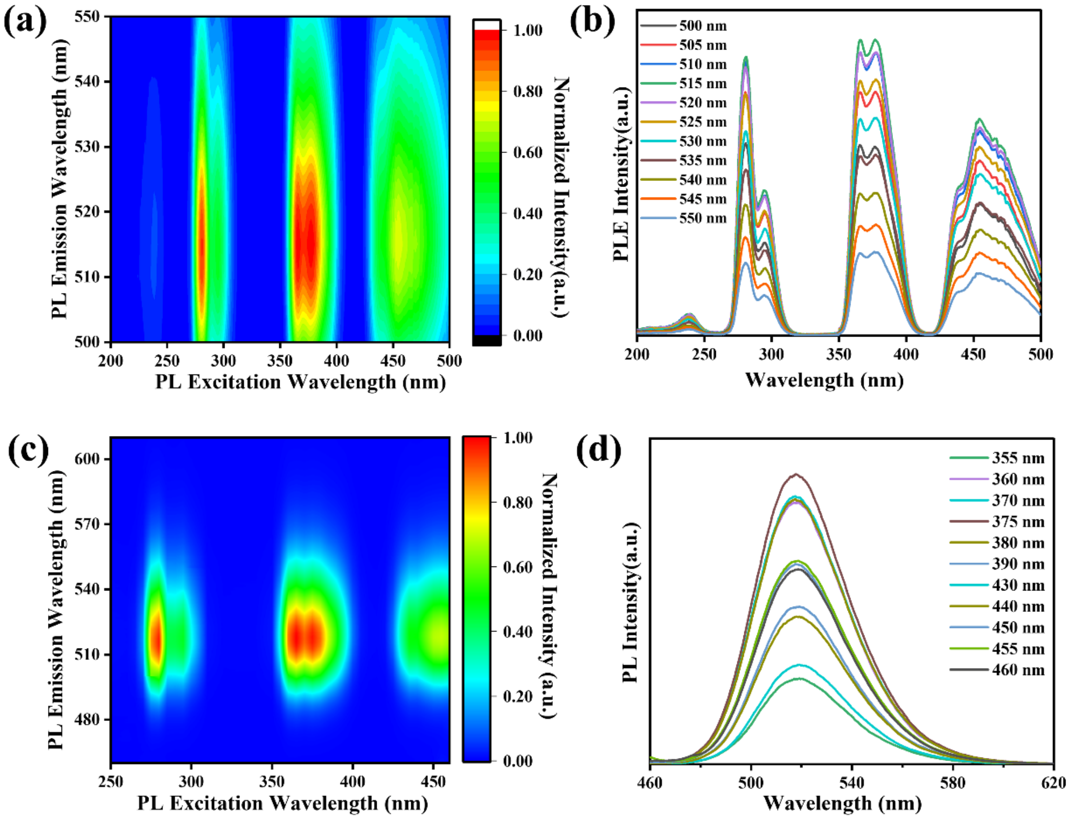

Figure S4. (a) Pseudo color mapping of PL emission dependent PL excitation spectra of $[(C_2H_5)_4N]_2MnBr_4$ MCs, (b) PL excitation spectra of $[(C_2H_5)_4N]_2MnBr_4$ MCs under different emission wavelength, (c) Pseudo color mapping of PL excitation dependent PL emission spectra of $[(C_2H_5)_4N]_2MnBr_4$ MCs, (d) PL emission spectra of $[(C_2H_5)_4N]_2MnBr_4$ MCs under different excitation wavelength.

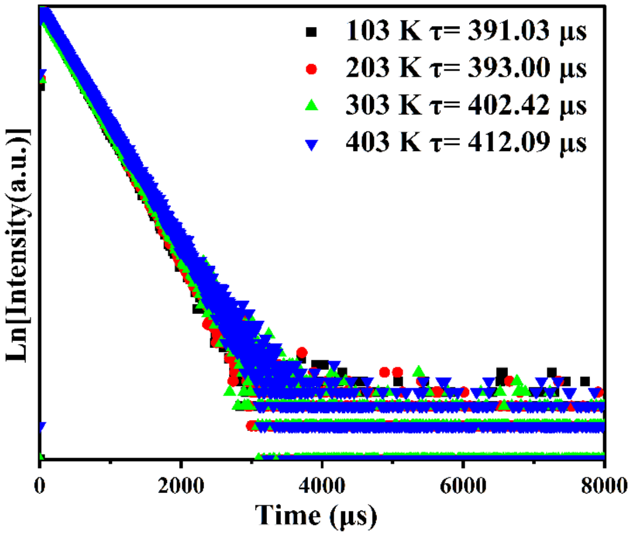

Figure S5. Temperature-dependent PL decay of $[(C_2H_5)_4N]_2MnBr_4$ MCs.

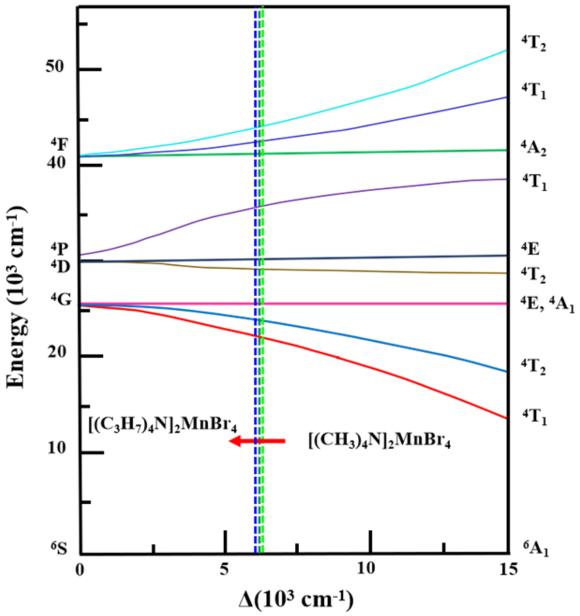

Figure S6. Tanabe-Sugano energy diagram of $3d^5$ electron configuration.

The calculations of crystal field (Δ) are based on Tanabe-Sugano energy diagram of d^5 electronic configurations, which can be obtained by the following equation (Eqs. (S1)–(S4)):

$$\Delta=10D_q \tag{S1}$$

$$^6A_1(^6S) \rightarrow [^4E(^4G),\ ^4A_1(^4G)] = 10B+5C+20\alpha \tag{S2}$$

$$^6A_1(^6S) \rightarrow {}^4E(^4D) = 17B+5C+6\alpha \tag{S3}$$

$$^6A_1(^6S) \rightarrow {}^4T_2(^4D) = 13B+5C+8\alpha \tag{S4}$$

$$^6A_1(^6S) \rightarrow {}^4T_2(^4G) = -10D_q+18B+6C-(26B^2/10D_q)+22\alpha \tag{S5}$$

The $^6A_1(^6S) \rightarrow [^4E(^4G), {}^4A_1(^4G)]$, $^6A_1(^6S) \rightarrow {}^4E(^4D)$, $^6A_1(^6S) \rightarrow {}^4T_2(^4D)$ and $^6A_1(^6S) \rightarrow {}^4T_2(^4G)$ can be obtained via converting the PLE into the diagram of energy coordinates.

For $[(C_2H_5)_4N]_2MnBr_4$, four excitation bands in the PLE spectrum, corresponding to the $^6A_1(^6S) \rightarrow [^4E(^4G), {}^4A_1(^4G)]$, $^4E(^4D)$, $^4T_2(^4D)$ and $^4T_2(^4G)$ transitions, were converted into energy

coordinates of 22727 cm^{-1} (440 nm), 26455 cm^{-1} (378 nm), 25674 cm^{-1} (389.5 nm) and 22026 cm^{-1} (454 nm), respectively. The crystal-field and Racah parameters were calculated to be Dq = 616.41 cm^{-1}, B = 82.81 cm^{-1}, C = 5279.29 cm^{-1}, $\alpha = -224.88$ cm^{-1} and $\Delta = 6164.12$ cm^{-1}.

For $[(C_3H_7)_4N]_2MnBr_4$, the four excitation bands assigned to the $^6A_1(^6S) \rightarrow [^4E(^4G), {}^4A_1(^4G)]$, $^4E(^4D)$, $^4T_2(^4D)$ and $^4T_2(^4G)$ transitions were converted into energy coordinates of 22831 cm^{-1} (438 nm), 26455 cm^{-1} (378 nm), 25773 cm^{-1} (388 nm) and 22124 cm^{-1} (452 nm), respectively. The corresponding parameters were calculated as Dq = 605.19 cm^{-1}, B = 54.78 cm^{-1}, C = 5382.51 cm^{-1}, $\alpha = -231.47$ cm^{-1} and $\Delta = 6051.96$ cm^{-1}. In comparison, the values calculated for $[(CH_3)_4N]_2MnBr_4$ are Dq = 628.57 cm^{-1}, B = 113.37 cm^{-1}, C = 5161.41 cm^{-1}, $\alpha = -213.24$ cm^{-1} and $\Delta = 6285.67$ cm^{-1}.

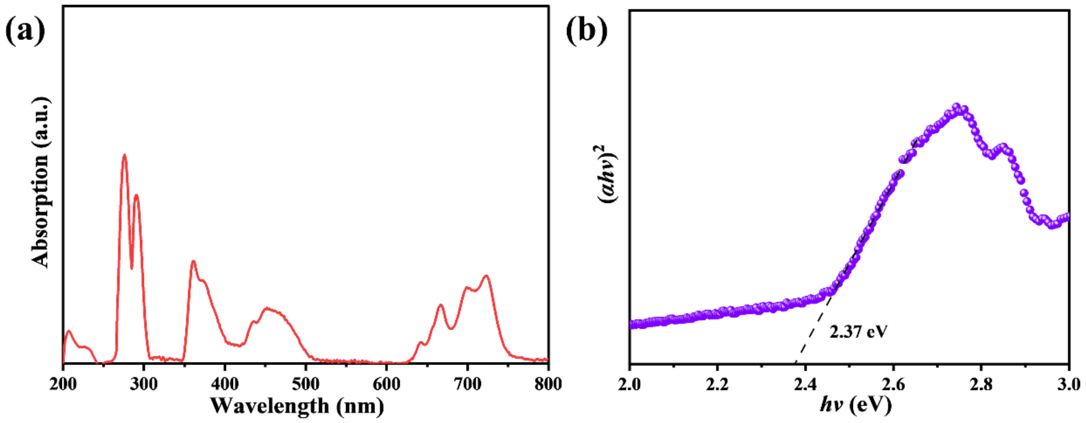

Figure S7. (a) Absorption spectrum and (b) corresponding optical band gap of $[(C_2H_5)_4N]_2MnBr_4$ MCs.

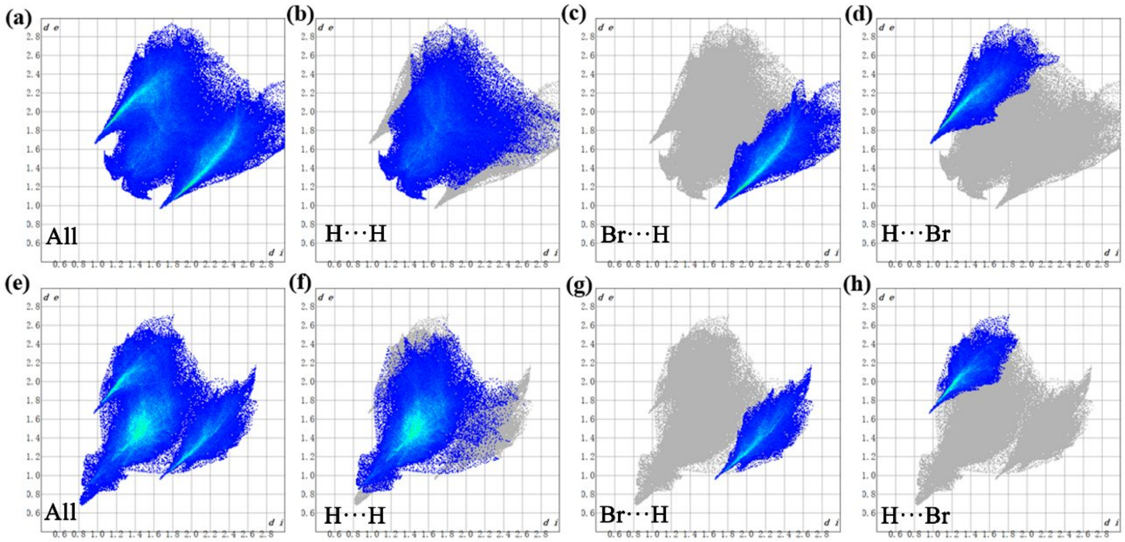

Figure S8. Fingerprint plots of $[(CH_3)_4N]_2MnBr_4$ and $[(C_3H_7)_4N]_2MnBr_4$: (a, e): all, (b, f) H···H, (c, g) Br···H and (d, h) H···Br interactions.

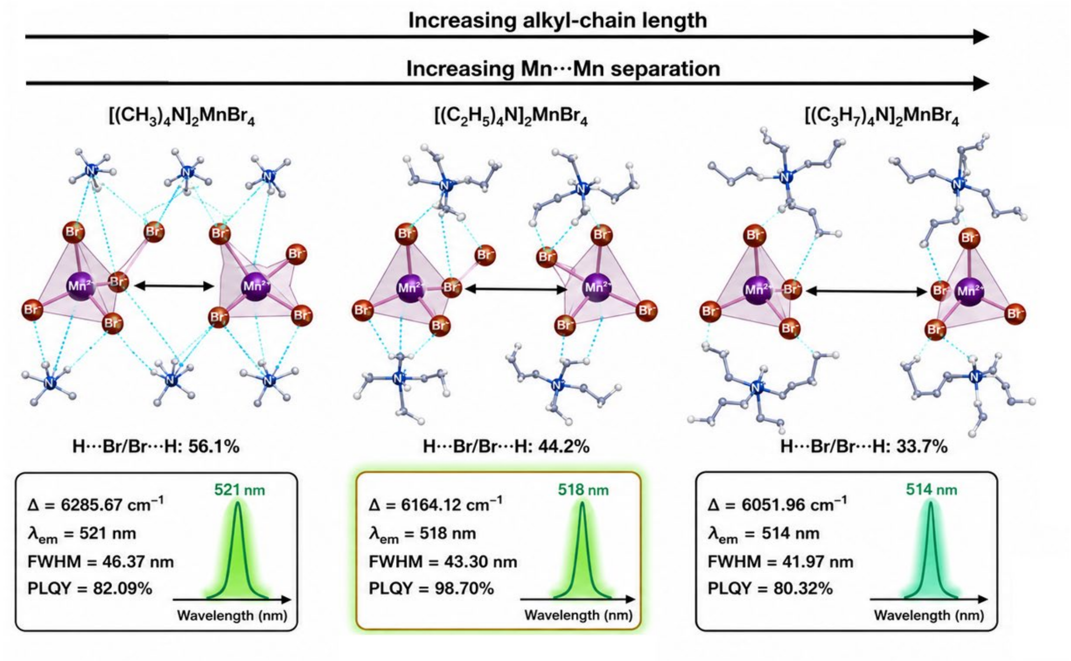

Figure S9. Schematic illustration of the relationship among organic-cation alkyl-chain length, Mn···Mn separation, intermolecular interactions, and photoluminescence properties of $[(CH_3)_4N]_2MnBr_4$, $[(C_2H_5)_4N]_2MnBr_4$, and $[(C_3H_7)_4N]_2MnBr_4$ MCs.

Table S1. Crystal data and refinement results of $[(C_2H_5)_4N]_2MnBr_4$ via Rietveld refinement.

Atom	Position	*x*	*y*	*z*	Ueq(Å^2)	Occupancy
Mn1	4e	0.748643	0.248645	0.230108	0.04344	1
Br1	4e	0.868080	0.368082	0.303138	0.04292	1
Br2	4e	0.755830	0.255832	0.051617	0.03119	1
Br3	8f	0.573130	0.290138	0.275647	0.03904	1
N1	2a	0.500000	0.50000	0.000000	0.02815	1
N2	4e	0.790235	0.709765	0.421767	0.01901	1
N3	8f	1.002936	0.505242	-0.003333	0.02858	0.25
Space group: P-$\bar{4}2_1$m (No. 113), tetragonal, Z=2, $\alpha=\beta=\gamma$=90°						
Cell parameters	a=13.370275 Å、b=13.37027 Å、.c=14.422973 Å and V=2578.313 Å^3					
Reliability factor:	R_{wp}=7.31%, R_p=5.54%, χ^2=2.88					

Table S2. Gibbs free energy changes of the products and reactants of the reaction.

$G_{product}$ (kcal/mol)	$G_{reactant}$ (kcal/mol)	ΔG (kcal/mol)
-7648677.70	-7648635.21	-42.49

Table S3. Crystal data and refinement results of $[(C_3H_7)_4N]_2MnBr_4$ via Rietveld refinement.

Atom	Position	*x*	*y*	*z*	Ueq(Å^2)	Occupancy
Mn1	8f	0.370452	0.250940	0.393430	0.01634	1
Br1	8f	0.422437	0.341551	0.333926	0.01158	1
Br2	8f	0.406043	0.116289	0.495470	0.01791	1
Br3	8f	0.344503	0.355379	0.495850	0.01728	1
Br4	8f	0.305173	0.192589	0.258990	0.01755	1
N1	4e	0.500000	0.909051	0.750000	0.01825	1
N2	8f	0.748467	0.514151	0.912008	0.00953	1
N3	4e	0.500000	0.413851	0.750000	0.00928	1
Space group: C12/c1 (No. 15), monoclinic, Z=8, α=γ=90°, β= 110.810°						
Cell parameters	a=33.352104 Å、b=14.372535 Å、c=15.072502 Å and V=6753.715 Å^3					
Reliability factor:	R_{wp}=7.07%, R_p=5.43%, χ^2=2.40					

Table S4. Interaction force contribution distribution of hybrid Mn^{2+} halides with different chain length in Hirshfeld surfaces.

Materials	H···H	H···Br/Br···H	Mn···H/H···Mn	Other
$[(CH_3)_4N]_2MnBr_4$	43.4%	24.1%/32.0%	0.2%/0.3%	0
$[(C_2H_5)_4N]_2MnBr_4$	54.6%	42.7%/1.5%	0/1.2%	0
$[(C_3H_7)_4N]_2MnBr_4$	64.1%	15.3%/18.4%	0.5%/0.7%	1%

Table S5. Comparison of green-emitting Mn^{2+} based hybrid halide in different organic cation.

Material	Synthesis	PLQY	λem (nm)	FWHM (nm)	Thermal Stability	WLED	Ref.
$C_{16}H_{38}N_2MnBr_4$ SC $C_{16}H_{38}N_2$=Decamethonium	Slow evaporation crystallization	76% @ 460 nm	530	~	~	LE=133 lm/W 108% of NTSC 1931	1
$(C_{20}H_{20}P)_2MnBr_4$ MC	Ultrafast self-assembly	93.83%@ 450 nm	523	48	83.5% of the initial intensity at 373 K	LE=117.17 lm/W 103.7% of the NTSC 1931	2
$(Meop)_2MnBr_4$ SC (Meop =1-(2-methoxyphenyl)piperazine)	Slow evaporation crystallization	99%@ 362 nm	521	41	93% of the initial intensity at 430 K	LE= 90.35 lm/W 105.7% of NTSC 1931	4
$(C_{22}H_{22}O_2P)_2MnBr_4$ MC (2-Ethoxy-2-oxoethyl)-triphenylphosphonium	Ball-milling method	96.1%@ UV light	520	45	77.3% of the initial at 120 °C	113% of the NTSC 1931	4
$(2\text{-}PI)_2MnCl_4$ SC (PI=2-phenylimidazoline)	Solution volatilization method	76.04%@ 314 nm	511	47.5	55.63% of the initial at 473 K	106.3% of the NTSC 1931	5
$[(CH_3)_4N][(C_2H_5)_4N]MnBr_4$ MC	Mechanochemical method	87.57%@ 455 nm	515	39.83	76.56% of the initial at 413 K)	LE= 78.16 lm/W 117.9% of NTSC 1931	6
$(4\text{-}BTP)_2MnBr_4$ SC (4-BTP=4-bromobenzyltriphenylphosphor	Slow evaporation crystallization	96.26%@ UV light	524	47.2	~	100.7% of NTSC 1931	7
$[Et(Ph)_3P]_2MnBr_4$ SC	Slow evaporation crystallization	94.4% @ 450 nm	523	50	47.3% of the initial at 165 °C.	LE=121.3 lm/W, 108.7% NTSC 1931	8
$[(C_2H_5)_4N]_2MnBr_4$ MC	Ultrafast self-assembly	98.70%@ 455nm	518	43.30	52.03% of the initial at 413 K	LE=134.60 lm/W, 112.6 % NTSC 1931	This work

Reference

(1) Zhang, S.; Zhao, Y.; Zhou, J.; Ming, H.; Wang, C.-H.; Jing, X.; Ye, S.; Zhang, Q. Structural Design Enables Highly-Efficient Green Emission with Preferable Blue Light Excitation from Zero-Dimensional Manganese (II) Hybrids. *Chem. Eng. J.* **2021**, *421*, 129886. doi: 10.1016/j.cej.2021.129886.

(2) Zhou, G.; Ren, Q.; Molokeev, M.S.; Zhou, Y.; Zhang, J.; Zhang, X.-M. Unraveling the Ultrafast Self-Assembly and Photoluminescence in Zero-Dimensional Mn^{2+} -Based Halides with Narrow-Band Green Emissions. *ACS Appl. Electron. Mater.* **2021**, *3*, 4144–4150, doi:10.1021/acsaelm.1c00606.

(3) Sun, C.; Zhang, S.; Wang, K.; Bai, S.; Tang, Z.; Su, B.; Xie, H. Highly Thermostable Hybrid Manganese Bromide as a Narrow-Band Green Phosphor for Backlight Displays. *Inorg. Chem.* **2026**. doi:10.1021/acs.inorgchem.6c02423.

(4) Meng, H.; Li, Y.; Zhu, M.; Chen, Y.; Shen, G. Facile Ball-Milling Synthesis of Highly Efficient Manganese Halides toward White Light-Emitting Display and X-Ray Imaging. *Sci. China Mater.* **2026**, *69*, 3273–3281. doi:10.1007/s40843-025-3886-1.

(5) Xiao, L.; Zhou, J.; Zhou, X.; Zhao, Y.; Tang, Z.; Xiong, T.; Wang, S.; Zhang, R. Zero-Dimensional Organic–Inorganic Hybrid Manganese Chloride with Narrow-Band Green-Emitting for Dual-Mode Backlight Display and X-Ray Imaging. *J. Lumin.* **2026**, *294*, 121884. doi:10.1016/j.jlumin.2026.121884.

(6) Tang, H.; Dai, T.; Tan, H.; Liu, H.; Kou, M.; Chen, Y.; Wu, Z.; Jiang, P.; Xiong, X.; Tang, Z.; et al. Double Organic-Ligand Enabled Narrow-Band Manganese Halides Toward Wide Gamut Displays, Flexible X-Ray Detection, and 3D Printing Applications. *Adv Funct Materials* **2026**, *36*, e25231, doi:10.1002/adfm.202525231.

(7) Yu, Y.; Liu, S.; Zhang, J.; Zhao, W.; Tang, Y.; Han, C.; Chen, X.; Xu, L.; Chen, R.; Li, M.; Tao, Y.; Lv, W. Mn(II)-Based Metal Halide with Near-Unity Quantum Yield for White LEDs and High-Resolution X-Ray Imaging. *Inorg. Chem.* **2024**, *63*, 10296–10303. doi:10.1021/acs.inorgchem.4c00978.

(8) Duan, J.; Yang, B.; Wang, D.; Wang, Y. Narrow-Band Green-Emitting Manganese(II) Hybrids Single Crystal with Record External Quantum Efficiency under Blue Light Excitation. *Laser Photonics Rev.* **2026**, *20*, e01442. doi:10.1002/lpor.202501442.
